# Interferon-λ Activates a Differential Response in Peripheral Neurons That Is Effective against Alpha Herpesvirus Infections

**DOI:** 10.3390/pathogens12091142

**Published:** 2023-09-07

**Authors:** Stephanie Salazar, Khanh T. Y. Luong, Taulima Nua, Orkide O. Koyuncu

**Affiliations:** Department of Microbiology and Molecular Genetics, School of Medicine and Center for Virus Research, University of California, Irvine, CA 92697, USA; ssalaza3@uci.edu (S.S.); luongkt1@uci.edu (K.T.Y.L.); tnua@uci.edu (T.N.)

**Keywords:** alpha herpesviruses, peripheral nervous system infection, interferon lambda, interferon stimulated genes, STAT activation

## Abstract

Alpha herpesviruses (α-HV) infect host mucosal epithelial cells prior to establishing a life-long latent infection in the peripheral nervous system. The initial spread of viral particles from mucosa to the nervous system and the role of intrinsic immune responses at this barrier is not well understood. Using primary neurons cultured in compartmentalized chambers, prior studies performed on Pseudorabies virus (PRV) have demonstrated that type I and type II interferons (IFNs) induce a local antiviral response in axons via distinct mechanisms leading to a reduction in viral particle transport to the neuronal nucleus. A new class of interferons known as type III IFNs has been shown to play an immediate role against viral infection in mucosal epithelial cells. However, the antiviral effects of type III IFNs within neurons during α-HV infection are largely unknown. In this study, we focused on elucidating the antiviral activity of type III IFN against PRV neuronal infection, and we compared the interferon-stimulated gene (ISGs) induction pattern in neurons to non-neuronal cells. We found that IFN pre-exposure of both primary neurons and fibroblast cells significantly reduces PRV virus yield, albeit by differential STAT activation and ISG induction patterns. Notably, we observed that type III IFNs trigger the expression of a subset of ISGs mainly through STAT1 activation to induce an antiviral state in primary peripheral neurons.

## 1. Introduction

Alpha herpesviruses (α-HV) are prevalent pathogens with approximately 70% of the adult population in the world infected with Herpes Simplex Virus-1 (HSV-1) alone. Herpesviruses infect the host via mucosal epithelial cells prior to establishing latency in the peripheral nervous system (PNS). Upon latency, periodic reactivation is observed with symptoms ranging from mild cold sores to keratitis causing potential blindness and may even lead to death of immunocompromised individuals in extreme cases, such as herpes encephalitis. Latency and reactivation are dependent upon the state of balance between viral factors, cellular signals, and innate and adaptive immune responses [1]. Although we learned the fundamentals of antiviral response against HSV-1 infections, there remains further understanding into the complexity of these virus–host interactions. Because of this knowledge gap, there is yet to be a therapy to halt reactivation from happening [2,3,4]. The commonly used antiviral drug, acyclovir, targets viral DNA replication, but it has no effect on the establishment of latency in the ganglia or preventing reactivations. As of now, there is no effective vaccine against either HSV-1 or HSV-2 [4,5]. More importantly, there seems to be a lack of mechanistic understanding of the effect of immune response elicited by infected epithelial cells on the spread of infection to the nervous system [6].

In α-HV infections, the mucosal tissue is the first target and represents the first line of defense against viral invasion. During this intrinsic immune response, cytokines are produced in response to foreign pathogens [7,8]. Epithelial cells line the tissues and cavities in the body forming a protective barrier against the environment and are the initial targets of infection. Epithelial cells activate autocrine and paracrine signaling upon viral invasion to induce apoptosis of the infected cells and alert nearby cells of the viral threat; however, if infection persists, then local and global innate and adaptive immune responses are induced. As such, innervating axons are exposed to proinflammatory and antiviral cytokines but are less likely to induce apoptosis of the ganglionic neurons.

Interferons (IFNs) are cytokines belonging to a multifaceted immune response against pathogens made and released by the host cell in response to invasion. There are three known types of IFNs: type I IFNs, type II IFNs, and type III IFNs. Type III IFNs, or interferon lambda (IFN-λ), is the latest addition to the IFN family, and are known as potent antiviral cytokines. IFN-λ consists of subsets known as IFN-λ 1-4 in humans whereas in mice there are two isoforms, IFN-λ2 and -λ3 [9,10]. Like type I IFNs, or IFN-α/β, IFN-λ has been shown to display similar mechanisms of induction following viral infection and is involved in innate immune response via induction of the Janus kinases-signal transducers and activators of transcription (JAK-STAT) pathway [9,10,11,12]. IFN-λ distinguishes itself by utilizing a different heterodimeric receptor complex known as interferon lambda receptor 1 (IFNLR1) and interleukin-10 (IL-10) receptor subunit [10,13]. Type I IFNs have been commonly shown to have higher acute immune response than type III in epithelial cells and organoids but not as highly induced in mucosal cells.

Recent studies have demonstrated that IFN-λ elicits a protective response against α-HV infection as opposed to type I IFNs in mucosal epithelial cells [14,15,16]. Mucosal epithelial cells are a subset of epithelia found within the respiratory, gastrointestinal, and genetical tracts with essential physiological functions [14,15,16]. Notably, type III IFNs have been shown to induce a lower systemic inflammatory response within mucosal epithelial cells as opposed to type I IFNs in various other virus infections [17,18]. During HSV-2 infection, both in vitro and in vivo studies have reported a tissue protective response against infection [19,20]. Importantly, type III IFNs have demonstrated potential restriction of HSV replication within the central nervous system [21,22]. Although it has been shown that type III IFNs have a tissue protecting antiviral effect in mucosal epithelial cells, studies focusing on the connection of the mucosa-nervous system barrier protection from viral invasion are lacking.

Prior studies have shown that type I and type II interferons (IFNs) induce a local antiviral response in axons via distinct mechanisms leading to a reduction in pseudorabies virus (PRV) particle transport to the neuronal nucleus [23]. Therefore, we hypothesized that the intrinsic and innate immunity at the peripheral tissues before the infection spreads to the nervous system plays a pivotal role in determining the latency and reactivation phenotype of the infection. Understanding the mucosal epithelium-nervous system junction and its barrier functions, particularly during viral infections, is pivotal in understanding the latency establishment and reactivation dynamics of α-HVs.

This study focuses on elucidating the antiviral activity of type III IFN in fibroblasts and primary neurons using Rat2 cells and embryonic rat superior cervical ganglionic (SCG) neurons. We have determined that both primary neurons and fibroblasts mount an antiviral response against α-HVs when treated with Type III IFN albeit with different mechanisms. Treatment of both primary neurons and Rat2 cells resulted in comparable phosphorylated STAT1 (p-STAT1) induction, whereas p-STAT2 activation was observed specifically in Rat2 cells. Comparative analysis of interferon stimulated genes (ISGs) further showed significant differences between neurons and Rat2 cells. Importantly, type III IFN pre-treatment led to a severe reduction in PRV virus yield as compared to type I IFN in Rat2 cells implicating its antiviral role in the mucosa-nervous system junction. These results are critical to determine the role of type III interferons in mucosal surfaces prior to viral invasion of the nervous system. Understanding the dynamic relationship between cell intrinsic responses of epithelia and nervous system will contribute to developing novel therapeutic approaches to culling α-HV latency establishment.

## 2. Materials and Methods

### 2.1. Cells and Virus Strains

Wild-type Rat2 cells were used as a fibroblast cell model (ATCC). The cells were cultured in Dulbecco Modified Eagle medium (DMEM) (Cytiva parent company Danaher Corporation, Washington, DC, USA), 1% penicillin-streptomycin (PS) (Cytiva parent company Danaher Corporation, Washington, DC, USA), and 10% fetal bovine serum (FBS) (Genessee, Burlington, MA, USA). Porcine kidney epithelial cell line (PK15) was grown in DMEM, PS, and 5% FBS. PRV-Becker is a wild-type laboratory strain [24]. PRV-180 expresses mRFP1-VP26 in a PRV-Becker background [25]. Viral titers were measured in PK15 cells after infecting Rat2 cells or neuronal cells, using the mentioned viral strains, respectively. Rat2 cell infections were carried out using multiplicity of infection (MOI) of 1, using PRV-Becker, while neuronal infections were performed using 10^5^ pfu (MOI 10) and 10^4^ pfu (MOI 1) of PRV-180 for 24 h prior to harvest and collection.

### 2.2. Primary Neuronal Culture

Superior cervical ganglion (SCG) in Campenot tri-chambers were used for all primary neuronal cultures 35mm tissue culture dishes (Corning, Corning, NR, USA) were coated with poly-DL-ornithine (Millipore Sigma, Burlington, MA, USA) and mouse laminin (Life Technologies Carlsbad, CA, USA) to promote neuronal adherence. SCG were isolated from 16- or 17-day Sprague Dawley rat embryos (Jackson Laboratories, Bar Harbor, MA, USA), as described by Curanovic et al. [26]. Sterile teflon Campenot tri-chambers were attached to coated 35 mm dishes with autoclaved silicone grease. The chambered dishes were filled with neurobasal medium (Gibco, Billings, MT, USA) + 50X B-27 supplement (Gibco, Billings, MT, USA) + 100X Penicillin-Streptomycin-Glutamine (Gibco, Billings, MT, USA) + 1000X murine nerve growth factor (Gibco, Billings, MT, USA). SCG were seeded in the S-compartments of the chambers. After two days, 1 μM Cytosine β-D-arabinofuranoside (Ara-C, Millipore Sigma, Burlington, MA, USA) was added to select against any mitotic cells. Two days after Ara-C addition, the medium was removed from chambers and replaced with fresh neuronal medium. Neuronal medium was changed every five to seven days. All animal work was performed in accordance with the Institutional Animal Care and Use Committee of the University of California, Irvine Research Board under protocol; AUP-21-008.

### 2.3. Viral Infection and Drug Treatment in Compartment Neuronal Cultures

To avoid any possible leakage from the soma (S) and neurite (N) compartments, 1% methylcellulose in neuronal media was placed in the middle (M) compartment for all experiments performed. Mouse IFN-λ2 (Millipore Sigma, Burlington, MA, USA, 200 ng/mL) or Rat IFN-β (Millipore Sigma, Burlington, MA, USA, 100 U/mL) were added to the S compartment 24 h prior to harvest or infection for a pre-treatment whereas post-treatments included placing IFN in 1 h post-infection. PRV-Becker or PRV-180 infections were conducted in S compartments and cells were harvested 24 hpi pre- or post-treatment conditions.

### 2.4. Western Blot Analysis

Cell lysates were prepared using RIPA-light buffer including 1 mM Dithiothreitol (DTT) and a protease inhibitor cocktail (Roche Holding AG, Basel, Switzereland). Following 30 min incubation on ice and sonication, cells were pelleted, and the supernatant was placed in a new tube with 5X Laemmli buffer before boiling at 95 °C. Immunoblotting was performed using anti-Beta-actin (1:10,000) (Millipore Sigma, Burlington, MA, USA), anti-pSTAT1 (1:2000) (Cell Signaling Technology, Danvers, MA, USA), anti-pSTAT2 (1:2000) (Millipore Sigma, Burlington, MA, USA), anti-Oas1 (1:2000) (Proteintech, Rosemont, IL, USA), anti-IFIT1 (1:2000) (Life Technologies Carlsbad, CA, USA), and anti-IRF9 (1:2000) (Proteintech, Rosemont, IL, USA). Secondary mouse or rabbit antibodies (Millipore Sigma, Burlington, MA, USA, 1:5000) were used following primary antibody incubation.

### 2.5. Real-Time Quantitative PCR

Neuronal cell bodies were collected from the S compartment of 3–5 chambers and total RNA was extracted using RNeasy Mini-Kit (Qiagen, Hilden, Germany) for both neurons and Rat2 cells. cDNA synthesis was carried out using SuperScript III (Life Technologies Carlsbad, CA, USA) first-strand synthesis kit with an oligo(dT) primer. A 96-well Type I IFN array (BioRad Laboratories, Hercules, CA, USA) containing 86 genes commonly induced by type I IFNs was performed in duplicate using neuronal cDNA. Kapa Sybr Fast qPCR (Roche Holding AG, Basel, Switzerland) master mix was prepared for RT-qPCR using Bio-Rad and Applied Biosystems RT-qPCR Thermocycler. Amplification was performed using the following conditions: 95 °C for 3 min, 40 cycles of 95 °C, 3 s, and 60 °C, 30 s. Following RT-qPCR of type I IFN array replicates, only 80 genes were analyzed using comparative Ct analysis. The following genes were not included in data analysis as they did not consistently pass the thresholds in replicate: Nos2, Socs1, Il10, Ccl5, Tnfsf10. GAPDH was used as the housekeeping gene to normalize Type I IFN array data.

Of these 80 genes, 22 genes of interest were chosen for the following analysis based on their normalized gene expression. Triplicates were prepared and fold changes were calculated using the comparative Ct method (2^−ddCt^) normalized to GAPDH in treated and untreated samples of both primary neurons and Rat2 cells. Primers were ordered from Integrated DNA Technologies (IDT, Coralville, IA, USA), as shown below in Table 1.

### 2.6. Statistical Analysis

An unpaired *t*-test was performed using GraphPad Prism 5.0. Construction of data plots and heatmaps were also created in GraphPad Prism 5.0.

## 3. Results

### 3.1. IFN-λ Treatment Activates a Differential Response in Rat2 Cells versus Primary Neurons

**Figure 1:**
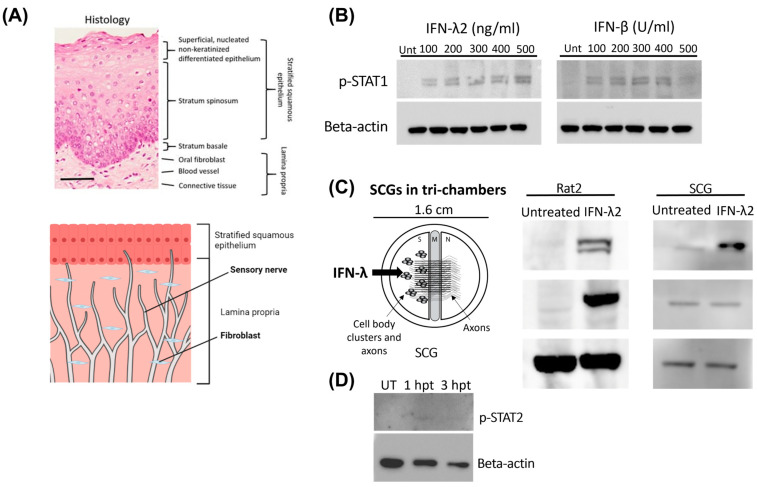
STAT activation upon IFN-β and IFN-λ2 treatment in Rat2 cells and SCGs. (**A**) Histological model of tissue from buccal oral mucosa adapted from Edmans et al., *Pharmaceutics*, 2020 (**top**) and a diagram model depicting fibroblasts and sensory nerves in the dermis (**bottom**) [30]. (**B**) Detection of p-STAT1 in Rat2 cells 24 h after treatment with either IFN-β or IFN-λ2 at various concentrations. (**C**) Model of treatment (**left**) and Western blots of detection of p-STAT1 or p-STAT2 upon IFN-λ2 (200 ng/mL) treatment for 24 h (**right**). (**D**) Detection of p-STAT2 in SCGs that were treated with IFN-λ2 (200 ng/mL) for 1 or 3 h (hpt) compared to untreated (UT) SCGs. Beta-actin was used as a loading control.

To determine whether treatment of primary neurons with type III IFN elicits a similar response to non-neuronal cells, superior cervical ganglionic (SCGs) neurons were compared to Rat2 cells (fibroblast cell line). SCG is the largest cervical sympathetic ganglia that innervates head and neck and has been shown to be a site for alpha herpesvirus latency [27,28]. Rat2 cells were chosen because of the positioning of fibroblasts within the connective tissue of the skin: they are found in two key areas known as the dermis (stratified squamous epithelia), which also hosts specialized immune cells, and the hypodermis (lamina propria), mostly consisting of adipocytes along with macrophages and axons of sensory neurons (Figure 1A). IFN-λ2 was utilized for the purpose of this study as it was shown to be conserved among humans, mice and rats, and to show higher antiviral activity in human and murine cells [29]. Rat2 cells were treated with either type I (IFN-β) or type III IFN (IFN-λ2) for 24 h prior to harvest and analysis (Figure 1B). STAT1 and STAT2 phosphorylation was monitored at different concentrations of IFN-λ2 and IFN-β treatment for comparative detection of phosphorylated STAT 1 (p-STAT1) (Figure 1B). Based on this data, in the subsequent experiments, we used IFN-λ2 at 200 ng/mL, and IFN-β at 100 U/mL concentration. Rat2 cells and neuronal cell bodies and axons within the S-compartment were harvested after 24 h IFN treatments to determine the phosphorylation status of STAT1 and STAT2, key mediators of the JAK-STAT signaling pathway by using p-STAT1 and p-STAT2 specific antibodies. Both cell types demonstrated comparatively increased levels of p-STAT1 when they were treated with IFN-λ2 as opposed to their untreated counterparts (Figure 1C). However, IFN-λ2-treated Rat2 cells demonstrated a much higher STAT2 phosphorylation when compared to SCG neurons (Figure 1C). When we monitored STAT2 activation in neurons at earlier time points after IFN-λ2 treatment (1- and 3 h post treatment), we did not detect any pSTAT2 accumulation (Figure 1D). Together, these results indicate that there is a cell-type specific induction of the signaling cascade upon exposure to type III IFNs.

### 3.2. IFN-λ Induces Differential Expression of Interferon Stimulated Genes (ISG) in Primary Neurons

**Figure 2:**
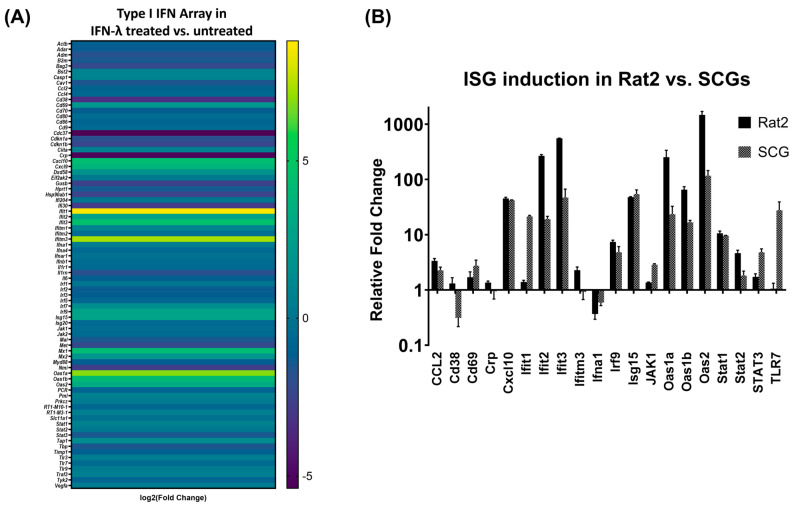
Comparative ISG induction upon IFN-λ2 treatment in Rat2 cells and SCGs. (**A**) SCGs were treated with IFN-λ2 or vehicle. Type I IFN array including primer pairs for 85 Interferon stimulated genes (ISG) was used (GAPDH was used as housekeeping gene). Heatmap created using Graphpad Prism 5.0 and performing log2 fold change on normalized gene expression (2^−ddCt^). Yellow represents higher fold change whereas blue indicates decreased fold change. (**B**) Normalized gene expression (2^−ddCt^) in SCGs (grey pattern) and Rat2 cells (black) treated with IFN-λ2 (200 ng/mL) for 24 h prior to harvest.

Interferons activate a signaling cascade that induces the expression of interferon stimulated genes (ISGs) which elicit protective responses against a viral threat. ISGs are key regulators of the JAK-STAT pathway that can impact early and late stages of the virus life cycle [10,31,32]. STATs serve as transcriptional activators of ISGs. As shown in the previous data above, Rat2 cells and SCGs activate STAT1 and STAT2 differently. To differentiate between ISG induction in neurons, a type I IFN array was used on a 96-well RT-qPCR plate with 84 genes of interest and 2 housekeeping genes. SCGs were either treated or not treated with IFN-λ2 (200 ng/mL) for 24 h prior to harvest and their mRNA levels were analyzed via RT-qPCR (Figure 2A). A heat map was constructed based on the mean cycle threshold (Ct) values detected in duplicate control versus IFN-λ treatment conditions demonstrating the activation of a subset of ISGs dependent on exposure to type III IFN. Based on the changes in the Ct values, 21 genes of interest were selected for further investigation.

For these ISGs, we designed primers based on the target sequences in the type-I IFN array. We treated both SCGs and Rat2 cells with IFN-λ2 (200 ng/mL) prior to RNA isolation. This experiment was repeated three times with replicates. Normalized gene expression analyses demonstrated that type III IFN induces a similar response in fibroblasts and primary neurons with differential activation dynamics (Figure 2B). The magnitude of ISG induction was detected to be lower overall in SCGs as compared to Rat2 expression. In SCGs, the highest ISG induction was detected to reach approximately 117-fold in the case of Oas2. In Rat2 cells, Oas2 was also the most activated gene and displayed approximately 1400-fold induction upon IFN-λ treatment.

Six ISGs demonstrated a higher fold change in SCGs than Rat2 cells. Stat3, Ifit1, and TLR7 showed a higher induction in SCGs than Rat2 cells displaying a 2.8-, 15.4-, and 27.8-fold difference, respectively, when compared to induction in Rat2 cells. Cd69 and Isg15 showed a fold change below 1.5-fold (Figure 2B). Only one ISG; Cd38 demonstrated a decrease in IFN-λ2-treated SCGs as compared to an increase in IFN-λ2-treated Rat2 cells. Furthermore, we detected that STAT1 expression was induced similarly in both cell types showing approximately 10-fold induction, whereas STAT2 transcripts were detected to increase 4.6-fold in Rat2 cells and 1.8-fold in SCGs supporting our differential STAT2 phosphorylation data (Figure 1C).

### 3.3. Verification of Target ISG Induction in Rat2 Cells vs. Primary Neurons upon IFN-λ Treatment

**Figure 3:**
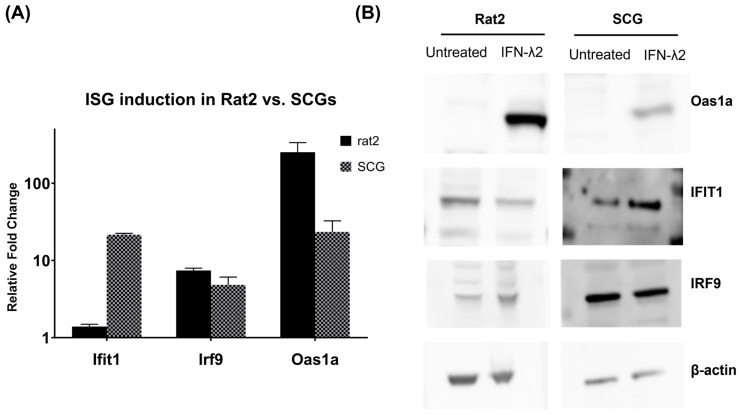
Cell-specific expression of genes of interest. (**A**) Normalized gene expression (2^−ddCt^) of Oas1a, IRF9, and IFIT1. (**B**) Western blots of Rat2 cells and SCGs untreated or treated with IFN-λ2 (200 ng/mL) for 24 h and immunoblotted with respective antibodies: Oas1a, IFIT1, and IRF9. Beta-actin was used as a loading control.

Three ISGs were selected for further protein analysis based on the differential gene expression data: Oas1a, IFIT1, and IRF9. Oas1a inhibits protein synthesis by inducing general RNA degradation within infected cells thus inhibiting viral replication along with IFIT1, which inhibits translation and viral replication. IRF9, a component of the complex known as IFN-stimulated gene factor 3 (ISGF3), is directly involved in the JAK-STAT pathway and is a key component in IFN immune response. These three genes showed a cell-type-specific difference in the induction pattern: Oas1a was detected to be increased in both cell types (at different magnitudes), Ifit1 showed a higher induction in SCGs, and Irf9 demonstrated a higher induction in Rat2 cells. Western blot analyses were performed on IFN-λ2-treated and untreated Rat2 cells and SCGs. Supporting our relative gene expression data shown in Figure 3A, Oas1a expression was detected to be increased in both cell types upon treatment of IFN-λ2, although at a higher level in Rat2 cells (Figure 3B). As expected, based on the transcriptional profile, IFN-λ2 (200 ng/mL)-treated Rat2 cells had lower activation of IFIT1 as opposed to SCGs which showed an increase in protein amounts (Figure 3B). Finally, IRF9 demonstrated a slight increase in Rat2 cells upon IFN-λ2 (200 ng/mL) treatment as opposed to untreated or IFN-λ-treated SCGs validating the transcriptional RT-qPCR analyses.

### 3.4. Antiviral Effect of IFN-λ in Rat2 Cells against Pseudorabies Virus (PRV)

Prior studies have demonstrated that both type I and type II IFNs exert antiviral activity against α-HV infection in both neuronal and non-neuronal cells [22,23]. To determine the antiviral potential of IFN-λ2 in Rat2 cells, we infected Rat2 cells with Pseudorabies virus Becker (PRV-Be), a wild-type strain of PRV. PRV is a member of the α-HV family that can infect many animals, and its replication cycle is similar to the human pathogen; HSV-1. Therefore, PRV has been used as a model α-HV in compartmentalized neuronal culture model [25,26,27,28,33,34].

**Figure 4:**
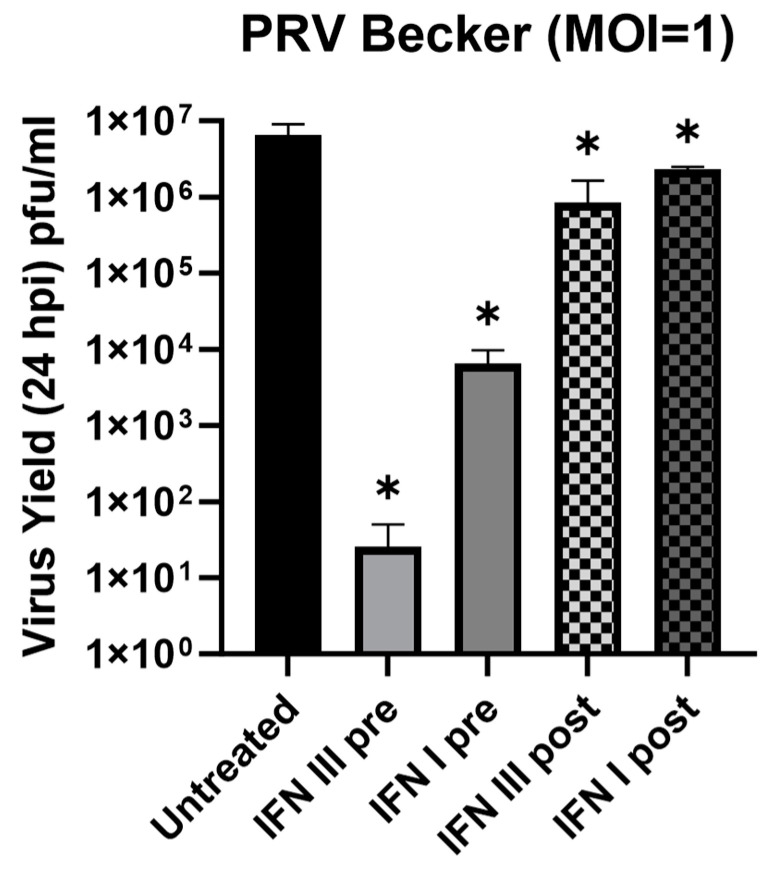
Antiviral effect of type III IFN in Rat2 cells. Rat2 cells pre- and post-treated with IFN-β (100 U/mL) or IFN-λ2 (200 ng/mL) for 24 h prior to infection PRV-Becker (MOI = 1). Harvest of these cells was performed 24 hpi. Statistical significance analyzed via Student’s *t* test (n ≥ 3) is indicated as follows: ns, not significant; *, *p* < 0.05.

We infected interferon-treated or untreated Rat2 cells with PRV-Be at an MOI of one for 24 h. Viral yields between treated and untreated cells infected with PRV were calculated to determine the protective effects of both pre- and post-treatments of type I and type III IFNs. Pre-treated cells were exposed to IFN-β (100 U/mL) or IFN-λ2 (200 ng/mL) for 24 h prior to infection with PRV. Post-treatments were carried out using the same IFN concentration 1 h after the infection. Cells that were treated with the IFN diluent (0.1% BSA in ddH_2_O) served as a control and the viral supernatants were collected at 24 h post infection (hpi). Virus yields were calculated as plaque-forming units (pfu) in PK15 cells. In non-treated control Rat2 cells, PRV infection yielded 6.6 × 10^6^ (±1.4 × 10^6^) pfu/mL progeny. We detected a significant reduction in the virus yields in the pre-treatment condition of both type I and type III IFN, with up to a 3-log decrease in IFN-β (6.5 × 10^3^ ± 1.8 × 10^3^ pfu/mL), and up to a 6-log decrease in IFN-λ2 pre-treatment (2.6 × 10^1^ ± 1.4 × 10^1^ pfu/mL) (Figure 4). Conversely, post-treatments with either interferon type showed a significant but modest reduction in the virus yield. IFN-λ2 and IFN-β post-treatments yielded 8.4 × 10^5^ ± 4.6 × 10^5^ pfu/mL and 2.3 × 10^6^ ± 1 × 10^5^ pfu/mL, respectively. This indicates that the introduction of exogenous IFNs (through paracrine signaling), before the viral particle invasion, primes cells for an antiviral response. If the virus infection is initiated prior to interferon exposure, the antiviral response of the host cell is less restrictive.

### 3.5. Antiviral Effect of IFN-λ in Primary SCG Neurons against Pseudorabies Virus (PRV)

**Figure 5:**
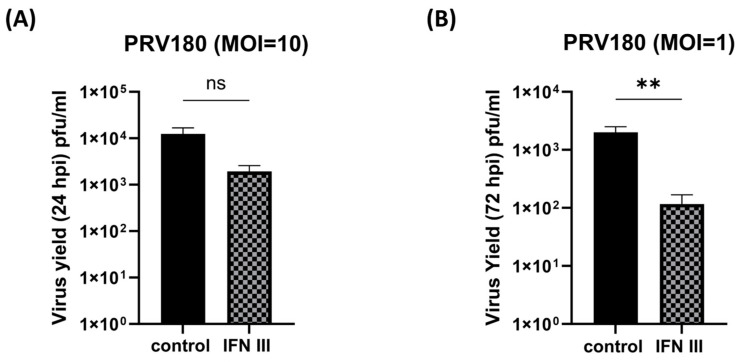
Antiviral effect of type III IFN in SCGs. (**A**) SCGs pre-treated with IFN-λ2 (200 ng/mL) for 24 h prior to infection with PRV-180 (10^5^ PFU) for 24 h. (**B**) SCGs pre-treated with IFN-λ2 (200 ng/mL) for 24 h prior to infection with PRV-180 (10^4^ PFU) for 24 h. Statistical significance analyzed via Student’s *t* test (n ≥ 3) is indicated as follows: ns, not significant; **, *p* < 0.005.

A similar approach was followed using primary SCG neurons to determine the antiviral potential of IFN-λ2 pre-treatment. In these experiments, we used PRV-180, a red capsid recombinant of PRV-Becker (mRFP-VP26) that allows for fluorescent imaging and observation of infected cells prior to harvest [25,26,27,28,33,34]. First, we infected SCGs at a high MOI (MOI 10) since PRV productive infections initiate efficiently at this MOI in primary neurons. The SCGs were treated with IFN-λ2 (200 ng/mL) in the S-compartment prior to infection for 24 h. SCGs were infected with PRV-180 (10^5^ pfu, MOI 10) in the S-compartment for 24 h before harvest (Figure 5A). Virus yields demonstrated a up to 10-fold decrease in IFN-λ2 pre-treated SCGs but did not show statistical significance (Figure 5A). In untreated SCGs, PRV-180 infection resulted in 1.2 × 10^4^ ± 4.3 × 10^3^ pfu/mL, and IFN-λ2 pre-treated SCGs yielded 1.9 × 10^3^ ± 6.4 × 10^2^ pfu/mL of infectious progeny in 24 hpi.

To investigate the effect of IFN-λ2 against a lower dose of PRV infection, we infected the neurons with PRV-180 at an MOI of 1 (10^4^ pfu), after a pre-treatment with IFN-λ2 (200 ng/mL). We detected a significant reduction in the virus yield in IFN-λ2 pre-treated samples (Figure 5B). At the MOI of 1, control SCGs yielded 2 × 10^3^ ± 4.9 × 10^2^ pfu/mL, whereas IFN-λ2 pre-treated neurons yielded 1.2 × 10^2^ ± 5.1 × 10^1^ pfu/mL virus with a 17-fold mean difference implying that the antiviral effect of type III IFNs in primary neurons is more significant at low MOI infections.

## 4. Discussion

Alpha herpesviruses establish latency within the host by entering the peripheral nervous system, causing a lifelong quiescent infection in the host. α-HV particles travel along the axons of the peripheral neurons before reaching to the neuronal nuclei to establish latency in the peripheral nervous system (PNS). During viral infection, proinflammatory and antiviral cytokines are induced; however, PNS neurons pose the dilemma of necessitating other means of protection. Cells undergoing infection of a pathogen commonly undergo an apoptotic response, but as PNS neurons are not capable of dividing and are thus limited to pro-survival mechanisms, other responses are vital for their protection [21,35,36,37]. Thus, the junction of mucosal epithelial cells and the nervous system represents a crucial barrier for the initial immune response that would prevent the viral spread to the nervous system while not activating apoptotic pathways in neurons.

Type I and type III IFNs share a similar signaling pathway known as the Janus kinase/signal transducer and activator of transcription (JAK-STAT) pathway. Both IFNs activate the phosphorylation of signal transducers and activators of transcription (STAT) 1 and STAT2 transcription factors via the JAK-STAT pathway. STAT1 and STAT2 are key mediators in interferon signaling and play a pivotal role in the JAK-STAT signaling cascade [38,39,40,41,42]. This pathway triggers the antiviral expression of interferon-stimulated genes (ISGs) upon detection of a virus infection and is involved in the intrinsic and innate immune response against viral infection. However, despite type I and type III IFN receptors converging on a similar signaling pathway, there are cell-specific differences in the expression of ISGs within the body. Previous studies have determined a major role of type I IFNs in most cell types, but it has only been recently reported that type III IFNs have antiviral effects on predominantly mucosal epithelial cells [18,19,43,44,45,46]. This infers that type III IFNs can be a restricted first-line defense against viral pathogens that invade the body via mucosae, but little is known about their role in a more general, systemic antiviral response.

**Figure 6:**
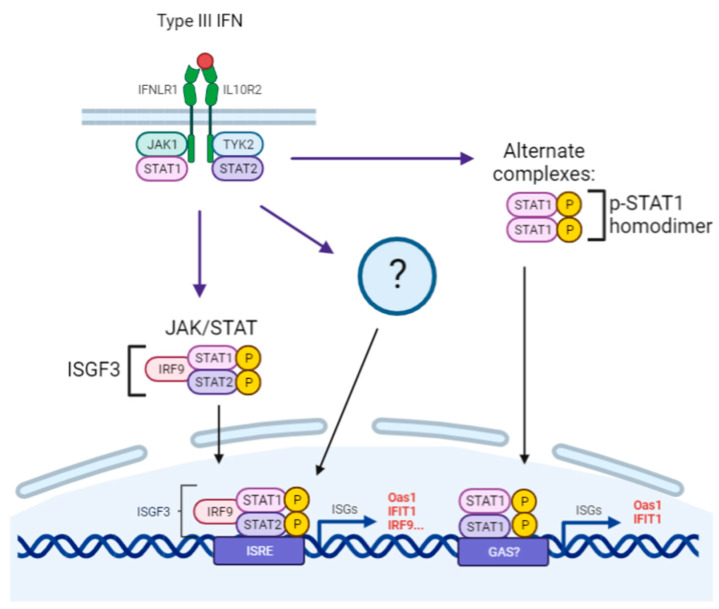
Model of canonical and alternative type III IFN signaling pathways. The model depicts classical and possible alternative pathway type III IFNs may induce in neurons. Type III IFNs may induce a downstream signaling involving p-STAT1 homodimers. These homodimers may bind to IFN-γ-activated site (GAS) elements instead of ISRE, inducing a unique subset of ISGs in a STAT1 dependent manner. Additionally, type III IFNs may activate unknown alternative pathways that induce ISG expression. Adapted from Biorender.

Due to their proximity to sensory neurons and their role within the dermis of the mucosal epithelia tissue, studying the signaling pathway within both fibroblasts and primary neurons provide comparative information on the connective tissue barrier between the initial infection site of α-HVs and the site of life-long latency. In this study, we show that STAT1 is phosphorylated upon type III IFN (IFN-λ) treatment both in primary peripheral neurons and fibroblasts (Rat2 cells), whereas p-STAT2 accumulation was detected only in fibroblasts. For both type I and type III IFNs, STAT1 and STAT2 bind to IRF9 and form a complex which binds to the IFN-stimulated response elements (ISREs) which in turn induce ISGs. This difference in phosphorylation of STAT2 in neuronal cell bodies may indicate a change in the overall intrinsic immune response within neurons as opposed to fibroblasts [21,37,39]. Further, these results indicate that although both cell types respond to type I and type III interferons, there is a distinction within the induction of the JAK-STAT signaling cascade upon IFN treatment. This could infer a non-canonical pathway that type III IFNs induce that is STAT1-dependent as an alternative to the classical signaling model shared with type I IFNs (Figure 6).

ISGs are key regulators of the JAK-STAT pathway that can impact the early and late stages of the virus life cycle. STATs serve as transcriptional activators of ISGs, and once they are localized to the nucleus, they drive the expression of these genes. By distinguishing which individual ISGs are significantly induced in either Rat2 cells or SCGs, we can identify cell-specific ISG induction patterns upon IFN treatment. Our current study revealed that the induction trend of a subset of ISGs upon type III IFN exposure in neurons and fibroblasts follows a similar trend, albeit with significant differences (Figure 2A). Notably, the magnitude of ISG transcriptional activation was approximately 10 times lower overall in primary neurons when compared to Rat2 cells; the average fold change in Rat2 cells is 136 as compared to 19.6 in neurons. We detected more than a 1400-fold induction in the case of Oas2 in Rat2 cells, while it was induced 117-fold in SCGs. Interestingly, we detected significant differences in STAT activation between the two cell types. While STAT1 expression was induced similarly in both cells, STAT2 showed a much higher activation in Rat2 cells, while STAT3 expression was induced 2.8-fold more in SCGs, demonstrating that type III IFN treatment elicits a different antiviral response in terminally differentiated and highly polarized neurons (Figure 2B).

Neuron-specific upregulation of STAT3 and TLR7 is not surprising, considering that both ISGs have been reported to have distinct roles in the nervous system. Among all STAT family members, STAT3 seems to be specifically associated with neuronal survival during development and after neuronal injury. Multiple growth factors, hormones, and cytokines signal through STAT3 to protect the nervous system against cell death by inducing pro-survival and repair genes [47]. These observations strongly suggest that STAT3 is a key factor in promoting neuronal survival. TLR7 has been known as an endosomal sensor of ssRNA viruses and synthetic oligonucleotides that signal through MyD88 to induce type I IFN and inflammation [48]. However, in the central nervous, system TLR7 agonists were shown to cause axonal injury, whereas they potentiated pain and itch sensation in the peripheral nervous system [49,50].

Prior studies have also demonstrated differences between type I and type III IFNs in terms of ISG induction kinetics: type III IFN-mediated expression moderately increases but persists over a longer period as opposed to a sharp increase during type I IFN-mediated induction that declines quickly over a shorter period of time [46]. This may serve to circumvent proinflammatory influx of cytokines at the site of mucosal infection that would otherwise negatively impact tissue connectivity and neuronal homeostasis.

We further analyzed the protein accumulation pattern of three ISGs to verify the differential gene expression results (Figure 3A). These ISGs showed a cell-type-specific difference in the induction pattern. Oas1a was detected to be increased in both cell types, whereas Ifit1 showed higher induction in SCGs while Irf9 showed a higher induction in Rat2 cells. Protein accumulation of these ISGs confirmed the transcriptional activation data. Oas1a was found to be highly enriched in both fibroblasts and primary neurons upon IFN treatment showing a more prominent increase in Rat2 cells. IFIT1 levels were detected to be higher in IFN-treated SCGs than Rat2 cells, and IRF9 showed a slight increase only in Rat2 cells supporting the gene expression analysis (Figure 3B). The differences in the magnitude and pattern of ISG activation can be attributed to the activation of only STAT1 in neurons upon IFN-λ2 treatment, as opposed to STAT1 and STAT2 activation in fibroblast. Although not fully understood in the literature, the difference in phosphorylation of STATs, a key component of the JAK-STAT pathway may indicate a non-canonical pathway in neurons.

To understand how these differences would affect the antiviral response of neurons and fibroblasts against α-HV, we tested the virus yield of the pseudorabies virus (PRV) in the absence and presence of IFN-λ in both primary neurons and SCGs. As shown in multiple studies on mucosal epithelial cells, type III IFN pre-treatment has a protective effect against different viruses [12,14,19]. Importantly, HSV-2 infection showed a reduced virus yield within genital mucosal epithelial cells both in vitro and in vivo when type III IFN is present during productive infection [19,51]. Here, we report that IFN-λ effectively suppresses α-HV infection in neurons and non-neuronal cells. IFN-λ pre-treatment dramatically reduced the virus yield in fibroblasts, even more than IFN-β pre-treatment. Interestingly, post-infection treatment failed to confer as significant of a reduction to pre-treatment which indicates that the introduction of exogenous IFN-λ before the initiation of infection is required to establish the antiviral state. If virus infection starts in the host cell, viral immediate early and early proteins are expressed which can counteract the action of host cell signaling (STAT) and effector (ISG) factors. In a physiological infection, such protection may be achieved through paracrine signaling when the IFNs secreted by the infected host cell bind to the receptors on the neighboring uninfected cells.

We found a similar antiviral response in primary neurons upon IFN-λ treatment. When we pre-treated SCGs with IFN-λ before infecting them with PRV at a high viral dose that is enough to infect all the cell bodies in the S-compartments (MOI 10) to start a productive infection, we found up to a 10-fold decrease in the virus yield. This antiviral effect was even more significant and potent when we infected SCGs at a low MOI (MOI 1) (Figure 5). Previous studies have shown that there is an infectious dose threshold to start productive versus latent infections in the peripheral neurons when axons are invaded by α-HV particles [52]. When axons of peripheral neurons were infected at high MOI (10 and 100) with PRV, virus particle transport in axons was efficient and productive infections could initiate at the neuronal cell bodies. Virus particle transport was detected to be less processive at an MOI of 1, which shows a delay in initiating productive infection in the cell bodies. Axonal infections with viral doses below 0.1 MOI were biased toward silenced, latent infections. We previously showed that the number of viral particles retrogradely moving toward neuronal soma is significantly reduced when these axons were pre-treated with type I and type II interferon favoring the establishment of latency [23]. All these observations suggest that when axons are exposed to IFN-λ, the predominant cytokine secreted by infected epithelia, an antiviral state is primed against α-HV neuroinvasion. How this local antiviral state affects the establishment of latency requires further investigation.

There is a major knowledge gap towards elucidating the regulation of α-HV spread from epithelial cells to peripheral neurons where latency is established. During productive α-HV infection, epithelial cells produce an influx of proinflammatory cytokines. It is highly possible that the release of cytokines alerts the surrounding cells, including the sensory nerves, which are near the mucosal epithelia. Our findings point to the possibility of communication between infected epithelia, fibroblasts, and peripheral nervous system neurons upon α-HV infection. Taken together, our findings demonstrate a cell-type specific induction pattern of ISGs upon type III IFN treatment. We showed that type III IFN exposure of fibroblasts or neurons prior to infection is sufficient to establish a potent antiviral state against α-HV infection via differential STAT signaling. Understanding the long-distance signaling upon type III IFN induction of peripheral neurons and their intrinsic response is key to determining the relationship between initial infection in epithelia and spread to the peripheral ganglia and will further contribute to developing therapeutic approaches against α-HV neuroinvasion.

## Figures and Tables

**Table 1:** Primer sequences of genes of interest.

Gene	Forward Primer	Reverse Primer
Actb	CGTCCACCCGCGAGTACAACC	CGACGACGAGCGCAGCGATATCG
CCL2	GCTAATGCATCCACTCTC	GTTTAACATTACTTAAGGC
Cd38	GCGTAGTCTTCATTGGTGATG	CGCTGACATCATCTTGGGACGC
Cd69	CGCAGTCTACAGAAGCAAC	GACTGAAACACTGGATTGGGC
Crp	GGGGCAGGAGCAGGACTCG	CATAGACTGCATTGATCTG
Cxcl10	GGCTTCCCAATTCTCTAAGAGC	GAGCAGGCTGGGGCATGGC
Gapdh	GCAACAATGTCCACTTTGTC	GCCTGGTTACCAGGGCTGC
Ifit1	CGCTACGCAGCCAAGTATTTCC	CTGTTGCTTGTAACAGAGCCC
Ifit2	GCTGAGTGCTTTGACACAGC	GCTCTCCAATGATTCCTTAC
Ifit3	GCAACTGCAGAATACAGG	CTTATTCATGTACTCCATAG
Ifitm3	CCCCCAGTGGCAGCGTTCACG	GAACAGTCCGACAGAGCC
Ifna1	GCCAGTAGGGAGTCTTCCTGG	GCTTGGGATGCAACCCTCC
Irf9	CCAACCTGCCACTCTCGC	CCTTGCACTTTCTCTTGGCC
Isg15	GGTCCCCTGAAACTAAGG	GCTGACCCAGTGAGTCTCTC
Jak1	CGAAGGCAGAAACTCCATG	CGGAACCTCTACCACGAGAAC
Oas1a	CGAATGTATCTCCCTGGGG	GCATAGACCGTGAGCAGC
Oas1b	GCGCCAATTCAGGAAGTACAGC	CCCAACCCACAATTCTACG
Oas2	GGTGATGATCGATGTGCTG	CTGGTCTTGGAACTGTTTG
Stat1	GCATTTAAAGTCATATTCATC	CCGTGATGTTAGATAAAC
Stat2	GGAGTGGGTGGGAAACGGG	GCAACATCTCCCACTGCGCC
Stat3	GCAGGAATCGGCTATACTGC	CCTGGCGCCTTGGATTGAGAGC
Tlr7	GGACTTCTTCAAGAATCTGC	GGCTAAGGAGAGAGTCGTTAG

## Data Availability

Not applicable.
